# Pathways driving the endocytosis of mutant and wild-type EGFR in cancer

**DOI:** 10.18632/oncoscience.67

**Published:** 2014-07-29

**Authors:** Kaia K. Hampton, Rolf J. Craven

**Affiliations:** ^1^ Department of Pharmacology and Nutritional Sciences, University of Kentucky, Lexington, KY

**Keywords:** signaling, endocytosis, receptor, kinase, therapeutics

## Abstract

EGFR (epidermal growth factor receptor) is activated through changes in expression or mutations in a number of tumors and is a driving force in cancer progression. EGFR is targeted by numerous inhibitors, including chimeric antibodies targeting the extracellular domain and small molecule kinase domain inhibitors. The kinase domain inhibitors are particularly active against mutant forms of the receptor, and subsequent mutations drive resistance to the inhibitors. Here, we review recent developments on the trafficking of wild-type and mutant EGFR, focusing on the roles of MIG6, SPRY2, ITSN, SHP2, S2R^PGRMC1^ and RAK. Some classes of EGFR regulators affect wild-type and mutant EGFR equally, while others are specific for either the wild-type or mutant form of the receptor. Below we summarize multiple signaling-associated pathways that are important in trafficking wild-type and mutant EGFR with the goal being stimulation of new approaches for targeting the distinct forms of the receptor.

## INTRODUCTION

### Regulation of wild-type EGFR trafficking

**Figure 1:**
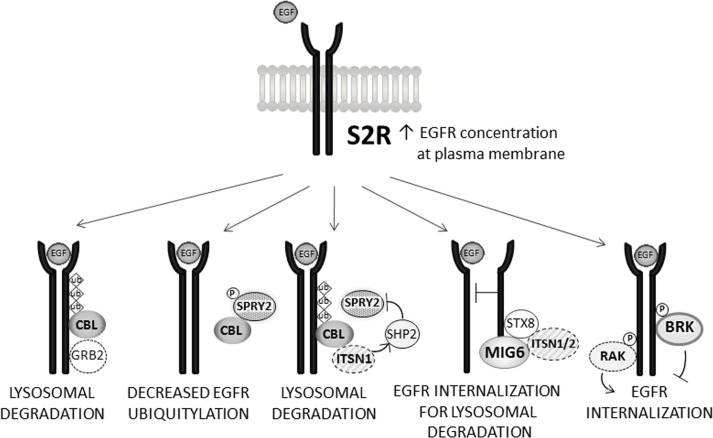
Selected pathways regulating EGFR endocytosis and degradation In the top panel, EGFR levels at the plasma membrane are increased by S2R^PGRMC1^. The diagrams, from left to right, below, show different binding partners for EGFR. GRB2 recruits CBL to EGFR resulting in lysosomal degradation. SPRY2 phosphorylation drives its association with CBL, inhibiting CBL binding to EGFR. ITSN1 can recruit SHP2 to dephosphorylate SPRY2, releasing CBL to bind EGFR. MIG6 physically obstructs EGFR dimerization and binds to STX8 and ITSN1/2 to promote lysosomal degradation of EGFR. BRK phosphorylates EGFR to inhibit EGFR internalization, while RAK/FRK has the opposite activity.

EGFR is over-expressed in a large number of tumors and is one of the best characterized oncogenic targets. EGFR binds to multiple extracellular growth factors, triggering conformational changes, dimerization of the receptor and phosphorylation of numerous residues in its cytoplasmic domain [1, 2]. Some of the phosphorylated sites serve as docking points for downstream signaling molecules, while others are bound by negative regulatory proteins that drive endocytosis of the receptor. Specifically, Cbl (Casitas B-lineage lymphoma), an E3 ubiquitin ligase [3], is recruited to tyrosine 1045 phosphorylated EGFR by the adaptor protein GRB2, which promotes EGFR ubiquitylation and entrance into clathrin coated pits (Figure 1 [4, 5]). There are a number of recent reviews on EGFR signaling and trafficking [6], so we will focus on several areas acting upstream on wild-type and mutant EGFR that have not been reviewed recently.

MIG6/RALT (mitogen-inducible gene/receptor-associated late transducer) inhibits EGFR [7-10] by associating with the receptor through a carboxy-terminal ERB-binding region (EBR). MIG6-EGFR binding physically obstructs EGFR asymmetric dimer formation [8, 11] and suppresses EGFR activity by stabilizing an inactive conformation of the receptor [12]. Many of these findings were reviewed in 2011 [13]. MIG6 decreases proliferation and migration in a variety of cell types *in vitro* [13]. In MIG6/*Errfi1* knockout mice, endogenous EGFR is hyper-activated, causing hyperplasia of epidermal keratinocytes and extreme sensitivity to chemical carcinogenesis [14] that is reversed by gefitinib.

More recently, Hopkins, *et al*., showed that mammary gland terminal end buds in *Errf1*-null mice had increased luminal filling [15]. This hyper-proliferation was not due to EGFR hyper-activation but decreased ABL activity, suppressing apoptosis in this setting [15]. ABL is a cytoplasmic tyrosine kinase associated with tumor cell survival and metastasis in cancer cells but also with stress-associated apoptosis through p73 in normal tissues [16-18]. MIG6 binds to ABL via its conserved ERB domain in the absence of epidermal growth factor, suggesting a mechanism in which MIG-6 senses EGF deprivation to induce apoptosis [15].

In addition to inhibiting EGFR catalytic activity, MIG6 increases EGFR internalization and trafficking to the lysosome [19], even for EGFR mutants that are not trafficked by CBL-mediated endocytosis [20]. Indeed, computational modeling suggests that MIG6 and CBL contribute equally to EGFR endocytosis [21], although this model is based on a limited number of cell lines. MIG6-dependent EGFR endocytosis is thought to be clathrin-dependent, involving binding between MIG6 and the SH3 domains of the intersectins ITSN1 and ITSN2 (Figure 1, [20]). Moreover, MIG6 associates with the SNARE protein syntaxin 8 (STX8, Figure 1), elevating levels of the STX8-EGFR complex, which is essential for EGFR endosomal trafficking [19]. The findings of Frosi, *et al*. suggests that clathrin-dependent EGFR endocytosis results in lysosomal degradation [20]. However, this model contrasts with a previous study indicating that clathrin-dependent EGFR endocytosis is associated with receptor recycling and sustained activation [22]. The latter study employed high levels of ligand, and the two studies were performed in very different cell types, HeLa [22] and mouse NR6 cells [20], implying that some aspects of EGFR endocytosis and signaling may be organism- or cell type-specific. Furthermore, there is recent biochemical evidence that MIG6 tyrosine phosphorylation weakens its ability to inhibit EGFR, even though the proteins remain associated [23]. Thus, the activation of various signaling pathways may have a profound effect on CBL function.

Although some studies suggest that MIG6 and CBL act through separate mechanisms, other findings suggest interplay between their EGFR endocytic pathways. Notably, intersectin 1 (ITSN1) forms complexes with both MIG6 and CBL, mediated by intersectin SH3 domains binding to the proline-rich carboxy-terminus of CBL [24] or proline-rich sequences located in the RED (RALT Endocytic Domain) of MIG6 [20], increasing repression of EGFR signaling [20] and EGFR ubiquitylation [24]. ITSN1, which has been reviewed recently [25], recruits other proteins downstream, particularly SHP2, SRC homology-2 containing phosphotyrosine phosphatase (Figure 1, [26, 27]).

SPRY2 is a CBL-binding protein [28] that can be tyrosine phosphorylated [29], driving its association with CBL and inhibiting CBL-RTK binding (Figure 1, [4]). SPRY2 is de-phosphorylated by SHP2, releasing CBL [30]. ITSN1 recruits SHP2 to SPRY2, disrupting the inhibitory effect of SPRY2 on CBL, promoting EGFR ubiquitylation and endocytosis (Figure 1, [26]). SPRY2 has been extensively reviewed elsewhere [31]. However, the role of the MIG6-ITSN complex in regulating CBL-SPRY complex formation is presently unknown. Thus, the interactions between EGFR, CBL, ITSN and MIG6 may be overlapping, perhaps to fine tune the temporal signaling through the receptor and to provide redundancy in the system.

### Sigma-2 receptor associates with EGFR, increases plasma membrane EGFR levels and promotes invasion

PGRMC1 (progesterone receptor membrane component 1) is a cytochrome b_5_-related protein that binds heme and is implicated in cellular trafficking [32]. There are compelling data that PGRMC1 is identical to the sigma-2 receptor (S2R). A highly selective S2R probe cross-linked directly to PGRMC1; S2R ligand binding decreased with PGRMC1-knockdown and increased with PGRMC1 over-expression; the apoptotic activity of an S2R ligand decreased with PGRMC1 knockdown; the PGRMC1 ligand AG-205 displaced S2R ligand binding [33, 34]. Notably, PGRMC1 was proposed to be a sigma receptor more than a decade earlier based on the ability of some sigma ligands to displace microsomal progesterone binding [35]. It is still formally possible that PGRMC1 is not itself the S2R but is part of a complex that is tightly associated with S2R, and numerous experiments are under way to further dissect this possibility. S2R^PGRMC1^ also plays a key role in membrane-associated progesterone signaling [36-38], but S2R^PGRMC1^ is not homologous to known steroid receptors and direct binding of progesterone to recombinant PGRMC1 has not been demonstrated. However, progesterone binding was detected to partially purified PGRMC1 [38], and RNAi inhibition of PGRMC1 decreased progesterone binding activity [38] suggesting that S2R^PGRMC1^ may influence progesterone signaling through a binding partner. Indeed, Thomas, *et al*. demonstrated that PGRMC1 forms a complex with mPRα and recruits the receptor to the plasma membrane [39]. Together, these proteins may be part of a larger membrane progesterone receptor complex.

In many peripheral tissues and in tumors, numerous groups have localized S2R^PGRMC1^ to the endoplasmic reticulum, endosomes, intracellular puncta and microsomal fractions [40-42]. Interestingly, S2R^PGRMC1^ localizes to a significant extent to the plasma membrane [43-45] and nucleus [46] in neuronal cells, and its interactions with receptors may occur at the plasma membrane in those tissues. Indeed, it is intriguing to speculate that S2R^PGRMC1^ might bind to a membrane progesterone receptor in neuronal tissues, where S2R^PGRMC1^ is co-expressed with membrane progesterone receptors [44], although this model is currently untested.

A number of groups have found that S2R^PGRMC1^ plays a profound role in regulating cellular signaling, particularly the Akt and ERK pathways [33, 40, 47, 48], and in searching for the mechanism underlying this effect, we found that S2R^PGRMC1^ associates with EGFR and co-localizes with EGFR within endosomes [40]. Furthermore, S2R^PGRMC1^ inhibition decreased plasma membrane levels of EGFR (Figure 1), and EGFR was de-stabilized by S2R^PGRMC1^ inhibition in some- but not all- cell types [40]. Thus, we propose a model that S2R^PGRMC1^ contributes to the trafficking of EGFR to the plasma membrane. An alternate model is that S2R^PGRMC1^ inhibits the endocytosis of EGFR, but S2R^PGRMC1^ was not detected at the plasma membrane in lung cancer cells [40], suggesting that any inhibition would be indirect.

Because S2R^PGRMC1^ associates with EGFR, we searched for downstream events regulated by this interaction and found that S2R^PGRMC1^ has a profound impact on protease activation in lung cancer cells [49]. Specifically, the S2R^PGRMC1^-EGFR complex increases the Lys310 acetylation and Ser535 phosphorylation of the NF-κB transcription factor, which in turn drives the expression of NGAL/LCN2 [49], a binding protein for matrix metalloproteinases such as MMP9 [50]. Indeed, MMP9 activity was largely dependent on S2R^PGRMC1^ expression in lung cancer cells. These activities required EGFR and were elevated by exogenous EGFR expression [49]. We note that other proteases, including MMP-2 and cathepsin D, were also activated in an S2R^PGRMC1^-dependent manner [49], and cathepsin D plays a key role in tumor invasion and metastasis [51].

S2R^PGRMC1^ is appealing as a cancer target because recent events suggest that it can be efficiently inhibited both by “PGRMC1” ligands, such as AG-205 [33, 52], and by a number of small molecule “sigma-2 receptor” ligands, including siramesine, PB28, SV119, CB-64D, SM-21 and others [53-58]. Some of these ligands have been extensively tested *in vitro*, *in vivo* and in clinical trials and had relatively minimal side effects. However, it is unclear whether any of these ligands alter EGFR trafficking. The interactions between these ligands and progesterone will likely reveal new elements of the S2R^PGRMC1^ mechanism.

### RAK/FRK increases EGFR trafficking

Our efforts in studying EGFR led to a second heretofore unknown pathway regulating EGFR trafficking. SRC family intracellular tyrosine kinases associate with growth factor receptors, including EGFR, and are important in mitogenic signaling through these receptors [59]. Indeed, SRC was the proto-typical oncogene, being mutated in transforming avian viruses. There are eight SRC-related tyrosine kinases with a common SH2 (SRC homology)-SH3 domain structure and a myristoylation site at the amino terminus [60]. The BRK/RAK/SRC42A/SRM kinases form a subgroup of proteins called the BRK family, that are related to SRC structurally but differ in the amino terminal sequences and multiple other sites [61]. In addition, the BRK/RAK/SRC42A/SRM proteins have widely divergent roles in cell proliferation.

While the majority of SRC-related kinases have a positive role on cell proliferation and survival, the RAK/FRK (FYN-related kinase [62, 63]) inhibits growth when expressed in a number of cancer cell types [64, 65]. RAK/FRK phosphorylates and binds to the PTEN tumor suppressor, stabilizing PTEN and promoting growth arrest, both *in vitro* and *in vivo* [66]. In addition, RAK/FRK associates with the RB (retinoblastoma) tumor suppressor [64] and phosphorylates a negative regulatory site on SRC [62]. Thus, there are multiple potential mechanisms through which RAK/FRK can inhibit cell growth.

We found that RAK phosphorylated tyrosine 1173 of EGFR and co-precipitated with EGFR [67]. The RAK-EGFR interaction required both the SH2 and SH3 domains of RAK and increased after EGF stimulation. As a result, RAK decreased the levels of EGFR at the plasma membrane (Figure 1, [67]), although it is unclear whether this was due to increased EGFR endocytosis, decreased EGFR transport to the plasma membrane, or some other factor.

Interestingly, BRK/PTK6 (breast tumor kinase/protein tyrosine kinase 6) also binds to EGFR and phosphorylates the receptor [68]. In addition, BRK phosphorylates CBL and promotes its degradation [69], potentially decreasing EGFR endocytosis (Figure 1). BRK/PTK6 drives breast tumor formation *in vivo* in mouse models [70, 71] and xenografts [71]. BRK is also a key effector of the MET receptor tyrosine kinase [71-73], and its stability is elevated by HER2 [74, 75].

Thus, two closely related RAK-BRK family members associate with EGFR, although their functions in the complex are opposite. The third family member, SRC42A, inhibits tyrosine kinase activity in *Drosophila* [76] in addition to playing a key role in development. SRM (SRC related kinase lacking C-terminal regulatory tyrosine and N-terminal myristylation sites) is induced in tumors and phosphorylates docking protein 11 [77] but does not perform an essential role in development [78], and any association with EGFR is unknown. It is intriguing to speculate that the EGFR-BRK and EGFR-RAK complexes (and possibly SRM or SRC42A complexes in *Drosophila*) may form in very different environments and with different dynamics during signaling.

### EGFR mutants drive tumor growth and have altered intracellular trafficking

Mutant forms of EGFR are associated with cancer development, including lung cancer in non-smokers, and with elevated sensitivity to EGFR inhibitors [79-83], such as erlotinib and gefitinib. It has become increasingly clear that distinct EGFR mutants have different patterns of regulation and trafficking (Table 1). For example, Furukawa*, et al*. reported that EGFR-Δ746-750 has sustained activation of downstream effectors and is not phosphorylated on Y1045, the CBL binding site, resulting in impaired endocytosis [84]. In the EGFR-L858R mutant, Y1045 is phosphorylated (81,82,83). However, in human cancer cells, EGFR-L858R is down-regulated [85, 86], but their assessment of ubiquitylation and CBL binding were markedly different [85, 86]. In contrast, Furukawa, *et al*., found normal EGFR-L858R-CBL binding with unaffected downstream signaling [84]. However, the latter studies were in mouse fibroblasts and simian COS-7 cells and may not reflect the signaling environment of cancer cells. The EGFR-L858R mutant has impaired nuclear EGFR localization resulting in decreased DNA repair activity [87]. Interestingly, the EGFRvIII mutant is also trafficked atypically, with the majority of the receptor being recycled to the plasma membrane rather than being degraded, even though the EGFRvIII mutation is on the extracellular surface of the protein [88].

**Table 1:** EGFR-L858R and EGFR-Δ746-750 mutants differ from wild-type EGFR in regulation and trafficking. Question marks indicate unknown, and a negative sign implies no effect

	Wild-type	L858R	Δ746-750
Endocytosis by MIG6	+++	+++	+++
Endocytosis by RAK	+	?	+++
Prevents endocytosis by SPRY2	+++	?	+++
S2R association	+++	?	+/−
Sensitivity to S2R inhibitor	+++	?	−
MIG6 Expression	+	++	?
MIG6 tyrosine phosphorylation	+	++	++
Impaired nuclear localization	−	+	?
CBL association	++	++	−

MIG6 expression is elevated in cells expressing EGFR-L858R [89], and MIG6 is required for the endocytosis of wild-type and mutant EGFR [89, 90]. However, MIG6 tyrosine phosphorylation is elevated in cells expressing EGFR-L858R and EGFR-Δ746-750 [91], suggesting a weaker ability to inhibit the receptor (Table 1). SPRY2 prevents endocytosis of both wild-type and Δ746-749/A750 mutant EGFR [90]. Thus, in a limited number of cell lines, MIG6 and SPRY2 do not discriminate between wild-type and mutant EGFR in their endocytic functions [90]. However, a recent study suggests that, although MIG6 is more efficient against the wild-type EGFR, it has an increased role in the endocytosis of EGFR Δ746-A750 compared to wild-type EGFR, because CBL is less active against the mutant [21]. The EGFR-vIII mutant does not undergo ligand-induced endocytosis due to low levels of phosphorylation [92], and MIG6 is inactive against it [19].

**Figure 2:**
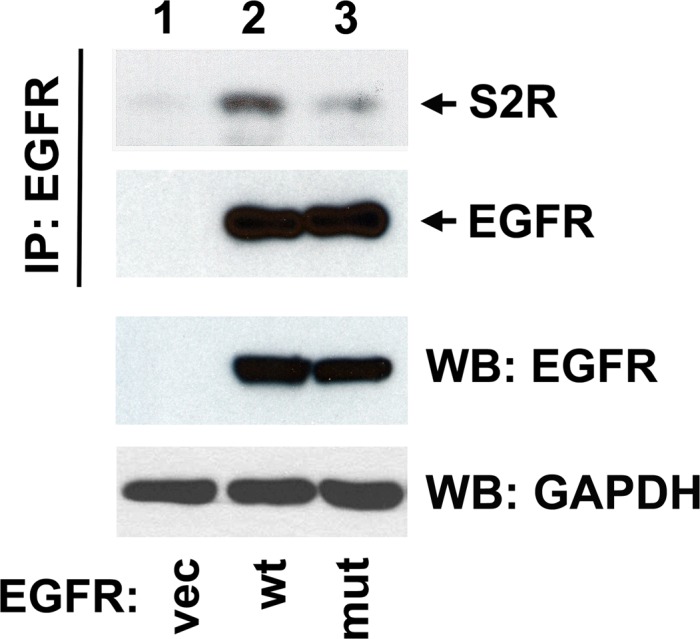
S2R^PGRMC1^ preferentially associates with wild-type EGFR MDA-MB-435 human breast cancer cells, which do not express EGFR (lower panel, lane 1), were transfected with a control plasmid (lane 1), the plasmid pcDNA3.1-EGFR (lane 2, a gift from Drs. Penni Black, University of Kentucky, and William Pao, Vanderbilt University) or the plasmid pBabe-EGFR-Δ746-749/A750P Addgene, Cambridge, MA). In the top two panels, lysates were immuno-precipitated using previously described conditions [40] with the anti-EGFR antibody IMC-C225 (Erbitux, ImClone Systems, Branchburg, NJ). Precipitates were then analyzed by western blot with (top panel) the anti-S2R^PGRMC1^ antibody PGR-UK1 [33] or (middle panel) an anti-EGFR polyclonal antibody (1005, Santa Cruz Biotechnology). Because of the very different molecular weights of the proteins, the blot was cut in half before probing. The bottom panels show the same unprecipitated cell lysates that were used for the precipitation reactions analyzed by western blot using EGFR and GAPDH polyclonal antibodies, the latter as a control for protein loading. The result shown is representative of three independent experiments. We have previously shown that the EGFR-Δ746-749/A750P mutant is highly tyrosine phosphorylated in this system compared to wild-type EGFR [67].

In contrast to MIG6 and SPRY, S2R^PGRMC1^ does not act equally on wild-type and mutant EGFR. An S2R^PGRMC1^ inhibitor was active against cells expressing wild-type EGFR, but had no activity against lung cancer cells expressing EGFR mutants [40]. However, the cell lines had different genetic backgrounds, and factors other than EGFR could have affected S2R^PGRMC1^ inhibitor sensitivity. For that reason, we expressed wild-type EGFR and the EGFR-Δ747-749/A750P mutant in MDA-MB-435 cells, which do not express EGFR, and found that S2R^PGRMC1^ co-precipitated with wild-type EGFR-2.4-fold more than the mutant (Fig 2). The mechanism underlying this specificity is unclear. However, S2R^PGRMC1^ is enriched in endosomes in lung cancer cells [40], and the decreased endocytosis of mutant EGFR may limit the access of the two receptors to each other. Because S2R^PGRMC1^ binds mutant EGFR poorly (and inhibitors are inactive against cells expressing it), we do not expect that S2R functions through a SPRY pathway for EGFR regulation.

In stark contrast to S2R^PGRMC1^, we found that RAK/FRK bound preferentially to the EGFR Δ746-749/A750P mutant compared to the wild-type protein [67]. This may be due to the increased activity of the mutant EGFR, because RAK binding to wild-type EGFR increased after ligand stimulation [67]. The results suggest that RAK may have elevated tumor suppressive activity in tissues expressing mutant EGFR. It is intriguing to speculate that tumors expressing mutant EGFR may have decreased RAK expression, but this concept has not been tested. Because RAK and MIG6 are both active against EGFR mutants, it is intriguing to speculate that they may be mechanistically related. However, this remains to be tested.

## PERSPECTIVES: CURRENT AND FUTURE WORK

Mutant forms of EGFR are associated with some types of cancer and have differential trafficking compared to the wild-type receptor. Furthermore, specific trafficking proteins are distinct in their regulation of wild-type and mutant EGFR. In spite of the dramatic advances in the field, there are numerous questions remaining about MIG6, such as the conditions under which its tyrosine phosphorylation changes and the key players directing these alterations. In addition, new targets for MIG6 play key roles in proliferation and apoptosis. In normal tissues lacking MIG6, breast cells proliferated due to loss of ABL [15], but it is unclear how the MIG6-ABL interaction changes in different non-malignant cell types and during cancer progression. In normal tissues, ABL is thought to be pro-apoptotic, while in cancer cells, ABL drives proliferation, survival and metastasis. But it is unknown whether ABL no longer binds MIG6 in cancer cells or whether binding changes in the presence of apoptotic stimuli.

The relatively poor binding of S2R^PGRMC1^ to the EGFR-Δ746-749/A750P mutant (Figure 2) may offer important clues to its interaction with EGFR. If the mutant is sustained at the plasma membrane, it is likely that S2R^PGRMC1^ is prevented from binding to mutant EGFR because EGFR is not internalized, consistent with the endosomal localization of S2R^PGRMC1^ in lung cancer cells [40]. For wild-type EGFR, EGF stimulation did not affect EGFR-S2R^PGRMC1^ binding [40], suggesting that the activated state of EGFR-Δ746-749/A750P is not responsible for the change in binding to S2R^PGRMC1^. Future work will include a broader analysis of S2R^PGRMC1^ binding to additional EGFR mutants, particularly EGFR-L858R.

The RAK/FRK tyrosine kinase decreases the plasma membrane pools of EGFR, and it is intriguing to speculate that RAK/FRK might influence the phosphorylation of EGFR trafficking proteins, including SPRY2, MIG6 and ITSN. We predict that RAK/FRK would increase MIG6 activity, possibly by decreasing MIG6 tyrosine phosphorylation. Conversely, we speculate that RAK/FRK might decrease the tyrosine phosphorylation of SPRY2, because SPRY2 phosphorylation is associated with decreased EGFR ubiquitylation (Figure 1). Current research is focusing on the role of RAK/FRK-PTEN binding in regulating EGFR.

## CONCLUSIONS

EGFR levels at the plasma membrane are balanced by competing positive and negative mediators. The impact of these pathways changes for the mutant forms of the receptor, and some of the regulatory proteins have altered expression in cancer. While kinase inhibitors are active against tumors expressing mutant EGFR, their activity is limited against those expressing wild-type EGFR, and some activities of EGFR may be kinase-independent. S2R^PGRMC1^ inhibitors are attractive in this setting because they inhibit EGFR-dependent cancer cell proliferation and are most active against the wild-type form of the protein [40].
